# Tomatoes protect against development of UV-induced keratinocyte carcinoma via metabolomic alterations

**DOI:** 10.1038/s41598-017-05568-7

**Published:** 2017-07-11

**Authors:** Jessica L. Cooperstone, Kathleen L. Tober, Ken M. Riedl, Matthew D. Teegarden, Morgan J. Cichon, David M. Francis, Steven J. Schwartz, Tatiana M. Oberyszyn

**Affiliations:** 1 Department of Food Science and Technology, The Ohio State University, Columbus, OH USA; 2 Department of Pathology, The Ohio State University Wexner Medical Center, Columbus, OH USA; 3 Department of Horticulture and Crop Sciences, The Ohio State University, Wooster, OH USA

## Abstract

Prolonged tomato consumption can mitigate ultraviolet (UV) light induced sunburn via unknown mechanisms. Dietary carotenoids distributed to skin are hypothesized to protect skin against UV-induced damage, although other phytochemicals may play a role. We hypothesize that tomato consumption would protect against skin cancer. SKH-1 hairless and immunocompetent mice (*n* = 180) were fed AIN-93G or AIN-93G + 10% *tangerine* or red tomato powder for 35 weeks. From weeks 11–20, mice (*n* = 120) were exposed to 2240 J/m^2^ UV-B light, 3x/week, and tumors were tracked weekly. Control mice were fed the same diets but not exposed to UV. Tumor number was significantly lower in male mice consuming red tomato diets (1.73 ± 0.50, *P* = 0.015) or pooled tomato diets (2.03 ± 0.45, *P* = 0.017) compared to controls (4.04 ± 0.65). Carotenoid levels in plasma and skin were quantitated, with total lycopene higher in skin of *tangerine* fed animals despite a lower dose. Metabolomic analyses elucidated compounds derived from tomato glycoalkaloids (including tomatidine and hydroxylated-tomatidine) as significantly different metabolites in skin after tomato exposure. Here, we describe that tomato consumption can modulate risk for keratinocyte carcinomas; however, the role of the newly identified specific phytochemicals possibly responsible for this action require further investigation.

## Introduction

Unprotected exposure to the sun is a major risk factor in the development of skin cancer^1^. Skin cancers, specifically keratinocyte carcinomas (KCs, often, but less accurately referred to as nonmelanoma skin cancers), are the most common of all cancers^2^, with more new cases (5.4 million in 2012) each year than breast, prostate, lung and colon cancers combined^3^. Despite a low mortality rate, KCs are costly ($8.1 billion/year), disfiguring, and rates are increasing^4^, prompting the U.S. Surgeon General to release a Call to Action to prevent skin cancer^5^. As a result, alternative methods for systemic protection, possibly via nutritional interventions to modulate risk for skin related diseases could provide significant benefit.

Human clinical data suggests that continued consumption of tomato paste can dampen UV-induced skin erythema (i.e., sunburn)^6^. It has been hypothesized that carotenoid pigments are the compounds responsible for this biological result, as one of the principal functions of carotenoids in plants is to act as photoprotectants^7^. In plants, carotenoids help to funnel energy away from chlorophyll and the photosynthetic apparatus, and can scavenge singlet oxygen^8, 9^. Following consumption, carotenoids are deposited in the skin of humans^10, 11^ where they are, in theory, present and able to protect from UV damage. Lycopene, the primary carotenoid in tomatoes, has been shown to be the most effective singlet oxygen quencher of the carotenoids^12^. However, when comparing lycopene administered from a whole food (tomato) or a synthesized supplement, tomatoes appear more efficacious in preventing redness after UV exposure, suggesting other phytochemicals in tomatoes may additionally contribute to this effect^13^.

The objective of this study was to determine whether dietary consumption of tomatoes (either as a *tangerine* or red variety), as compared to a tomato-free diet, could differentially reduce the UVB-induced tumor promotion and progression after chronic UVB exposure in male and female SKH-1 murine skin. We hypothesized that tomato consumption would decrease tumor number in animals consuming tomatoes, and that this biological effect would be the result of altered skin and plasma metabolomes.

## Results and Discussion

### Animal weights and skin fold thickness

All animals consumed the three diets and gained weight. Male mice were given diets to provide 5 g food/day while female were given 4 g/day, and mice were allowed to consume as much of this allotment as desired. Male mice were significantly heavier than female mice (*P* < 0.001). Within a sex, there were no significant differences in body weights of animals on the different diets, or with UV exposure. Others have also found that rodents who consume tomato diets are heavier than mice on purified AIN-93G diets [27] while some have found no difference [21]. Skin fold thickness 48 hours after the first exposure to UV radiation can be used as a rough measurement of the inflammation response. Male mice had significantly thicker skin than female mice (*P* < 0.001), with an average (± standard deviation) thickness of 1.23 ± 0.15 mm in males as compared to 1.02 ± 0.11 mm in females. This held true even in mice not exposed to UV denoting that there are fundamental differences in the physiology of skin between the sexes. There was no significant differences in skin fold thickness between the control and tomato diets for either sex after exposure to UV, consistent with previous reports from our group (data not shown)^14^.

### Carotenoid profile in tomato diets

The control, *tangerine* and red tomato diets were extracted and analyzed for carotenoids (Table 1). The control diet did not contain tomato or any carotenoids. Phytoene and phytofluene were present in approximately twice the concentration in the *tangerine* tomato diet compared to the red tomato diet. Total lycopene was approximately three times higher in the red tomato diet compared to the *tangerine* diet. About 1% of the lycopene in the *tangerine* tomato diet was present as all-*trans*-lycopene while 82% of the lycopene in the red tomato diet was present as all-*trans*. Overall, the *tangerine* tomato diet had approximately a 20% higher level of total carotenoids compared to the red diet. ζ-carotene, neurosporene and tetra-*cis*-lycopene were absent in the red tomato diet, and β-carotene was absent in the *tangerine* tomato diet. Diets were formulated based on equal weight addition of tomato powder and are therefore not matched for any carotenoid levels.

**Table 1:** Carotenoid composition of *tangerine* and red tomato fortified diets^a^.

Carotenoid	AIN-93G + 10% *tangerine* tomato (mg/kg feed)	AIN-93G + 10% red tomato (mg/kg feed)
Phytoene	64.1	37.0
Phytofluene	17.8	9.6
ζ-carotene	22.9	nd
Neurosporene	8.2	nd
Tetra-*cis*-lycopene	18.6	nd
Other-*cis*-lycopene	2.8	10.9
All-*trans*-lycopene	0.8	50.0
β-carotene	nd	4.6
Total lycopene	22.1	60.9
Total carotenoids	135.0	111.9

Nd: not detected. ^a^No carotenoids were detected in the control AIN-93G diet.

### Carotenoid levels in blood plasma and skin

Carotenoids exist in the plasma of mice fed both tomato diets in the mid-to-high nanomolar range, with lycopene being the most prevalent carotenoid present. Statistically significant differences in carotenoid levels between mice by UV status, sex and tomato diet are noted for plasma in Table 2 and for skin in Table 3. Phytoene and phytofluene were found to be significantly higher in the blood of mice fed *tangerine* tomatoes (P < 0.001 for both carotenoids), as well as higher in females as compared to males (P < 0.0001 and P = 0.0102, respectively). ζ-Carotene, neurosporene and tetra-*cis*-lycopene are absent in the animals fed red tomato diets (given they are absent in red tomatoes), and therefore significantly higher in animals fed *tangerine* tomato containing diets. Other-*cis*-lycopene is significantly higher in mice fed tangerine tomatoes (*P* < 0.001) while all-*trans*-lycopene (P < 0.0001) and 5-*cis*-lycopene (P < 0.0001) are higher in mice fed red tomato diets. However, there was no overall significant difference in total plasma lycopene concentrations between the two diets. Since there was approximately 3 times less total lycopene delivered in the *tangerine* tomato diet, which still resulted in similar plasma lycopene levels, lycopene from *tangerine* tomatoes appears to be considerably more bioavailable compared to red tomatoes. This is consistent with what our group has seen in post-prandial bioavailability studies in humans, with higher bioavailability of lycopene from *tangerine* tomatoes as compared to red^15^.

**Table 2:** Concentration of carotenoids in plasma of mice consuming *tangerine* and red tomato diets.

Carotenoid	Fed *tangerine* tomato diet nmol/L plasma (std. dev.)				Fed red tomato diet nmol/L plasma (std. dev.)				Statistically significant differences^a^
	Male		Female		Male		Female		
	No UV n = 9	UV n = 10	No UV n = 10	UV n = 10	No UV n = 8	UV n = 10	No UV n = 10	UV *n* = 10	
Phytoene	69.0 (28.8)	50.7 (19.3)	224.1 (96.2)	151.2 (67.4)	50.4 (19.0)	33.4 (13.5)	142.4 (56.6)	129.2 (64.1)	Sex, tomato, sex*tomato
Phytofluene	50.7 (16.3)	55.0 (21.7)	108.0 (48.8)	53.6 (26.6)	34.4 (16.6)	22.8 (12.3)	46.4 (26.4)	44.2 (14.4)	Sex, tomato
ζ-carotene	99.9 (42.2)	159.4 (66.8)	222.0 (87.1)	126.8 (79.9)	nd	nd	nd	nd	Tomato
Neurosporene	78.4 (34.5)	124.2 (55.7)	162.2 (64.3)	96.9 (54.5)	nd	nd	nd	nd	Tomato
Tetra-cis-lycopene	130.9 (87.7)	148.5 (105.2)	175.8 (108.5)	113.0 (79.1)	nd	nd	nd	nd	Tomato
Other-cis-lycopene	97.0 (49.8)	139.0 (64.9)	200.2 (88.9)	118.1 (68.8)	67.9 (24.0)	55.1 (15.2)	57.1 (13.3)	62.1 (23.0)	Tomato
All-trans-lycopene	29.6 (11.0)	52.6 (28.1)	66.4 (20.8)	36.8 (22.8)	224.8 (93.5)	186.2 (96.4)	178.2 (54.2)	188.5 (58.3)	Tomato
5-cis-lycopene	29.0 (13.6)	49.2 (21.0)	55.7 (17.3)	30.5 (19.2)	84.6 (23.4)	77.7 (35.5)	64.1 (15.7)	69.2 (25.2)	Tomato
Cis-lycopene	256.9 (142.5)	336.7 (187.9)	431.7 (204.1)	261.6 (165.3)	152.5 (46.2)	132.9 (49.7)	121.2 (27.7)	131.3 (47.5)	Tomato
Total lycopene	286.5 (150.7)	389.3 (215.4)	498.1 (220.3)	342.7 (184.0)	377.2 (138.5)	319.4 (144.9)	299.4 (77.6)	319.8 (103.9)	NSD

Nd: not detected. ^a^Indicates factors that had statistically significant differences amount groups after univariate modeling (*P* < 0.05).

**Table 3:** Concentration of carotenoids in murine skin fed diets containing *tangerine* and red tomato powders.

Carotenoid	Fed tangerine tomato diet pmol/g skin (std. dev.)				Fed red tomato diet pmol/g skin (std. dev.)				Statistically significant differences^a^
	Male		Female		Male		Female		
	No UV	UV	No UV	UV	No UV	UV	No UV	UV	
Phytoene	57.0 (20.5)	63.6 (30.0)	110.9 (56.2)	108.1 (31.8)	33.9 (17.3)	77.0 (47.2)	123.4 (34.1)	56.8 (26.5)	Sex, UV status*sex
Phytofluene	19.8 (4.0)	17.7 (6.3)	30.6 (11.6)	21.8 (7.9)	nq	nq	nq	nq	Tomato
ζ-carotene	185.2 (76.1)	362.1 (272.1)	998.5 (1108.6)	294.2 (111.3)	nd	nd	nd	nd	Tomato
Tetra-cis-lycopene	120.6 (64.4)	162.5 (81.4)	207.0 (106.2)	146.8 (40.7)	nd	nd	nd	nd	Tomato
All-trans + other-cis-lycopene	424.8 (178.6)	882.4 (708.9)	1772.9 (1708.3)	672.7 (266.1)	235.5 (261.6)	307.5 (123.6)	734.2 (599.1)	1081.7 (796.5)	Sex
Total lycopene	545.4 (241.7)	1044.8 (783.1)	1981.9 (1764.7)	881.2 (309.8)	235.5 (261.6)	307.5 (123.6)	734.2 (599.1)	1081.7 (796.5)	Sex, tomato

Nq: not quantifiable; nd: not detected. ^a^Indicates factors that had statistically significant differences amount groups after univariate modeling (*P* < 0.05).

Carotenoids were found in the skin of mice in mid-picomole to low nanomole per gram concentrations (Table 3). Phytoene, all-*trans*-lycopene plus other-*cis*-lycopene and total lycopene content in skin was affected by sex, with females accumulating higher levels than males. Phytofluene, ζ-carotene, tetra-*cis*-lycopene and total lycopene were significantly higher in mice fed *tangerine* tomato diets, while phytoene and all-*trans* plus other-*cis*-lycopene were not different between the two diets. Because of significant matrix suppression with quantiation by MS (signal too low for PDA) for many carotenoids using C30 stationary phases, we chose to move to a C18 stationary column, which chromatographed interfering skin lipids separately from carotenoids, allowing quantitation via MS without the ~10-fold matrix suppression observed using C30 columns. However, this move from C30 to C18 eliminated more detailed data about lycopene isomer profiles in the skin, as here we report tetra-*cis*-lycopene and a combination of all-*trans*-lycopene plus other non-tetra-*cis*, *cis*-lycopene isomers as compared to tetra-*cis*-lycopene, other-*cis*-lycopene, all-*trans*-lycopene and 5-*cis*-lycopene quantitated separately in the plasma.

Carotenoid levels in both plasma and skin were of similar magnitude as found by Kopec *et al*. where 10% *tangerine* tomato diets were fed for 10 weeks, mice were exposed to a single dose of UVB light (1 M.E.D.) and scarified 48 hours later^14^. Lycopene was the carotenoid present in highest concentration in both plasma and skin in both tomato diets, despite not being the most prevalent carotenoid in the *tangerine* tomato diet. Hata *et al*. found an average of 69 ng lycopene (129 pmol lycopene/g) and 65 ng phytoene (120 pmol phytoene/g) per gram skin, in the range of what we see in the SKH-1 mice. Ribaya-Mercado found approximately 1.5 nmol lycopene/g skin as determined using HPLC while Mayne *et al*. found from 100–800 ng carotenoids/g skin (approximately 186–1493 pmol carotenoid/g skin) as determined using Raman resonance spectroscopy^16, 17^. This data suggests that SKH-1 mice accumulate carotenoids in skin in reasonably similar concentrations as do humans, contrary to what is seen in blood plasma. Lycopene has been shown in cell culture to follow a U-shaped curve in terms of its benefit in fibroblasts exposed to UVB light. At optimal levels (0.05 nmol/mg protein), lycopene was shown to decrease UV-induced formation of thiobarbaturic acid-reactive substances (TBARS) to 40–50% the levels of controls, indicating protection against lipid peroxidation^18^. A study in hairless mice found that supplementation of diets with palm fruit carotenoids can decrease TBARS from controls^19^. There were no significant differences due to UV status, likely because carotenoids were measured at the end of study, 15 weeks after the last UV treatment. Others have found that UV radiation decreases carotenoids in skin (but not plasma) when skin is collected 48 hours after a one-time exposure^20^. A similar result has been observed in humans, where reductions in plasma carotenoids were noted after individuals were exposed to UVA and UVB light over a two week period^21^. The possibility exists that our skin samples were taken too long after the last UV exposure (15 weeks) and by this point, UV induced differences in carotenoid content in the skin or plasma are lost.

There was considerable inter-individual variability, consistent with the use of an outbred strain of mouse. Large heterogeneity is consistent with human populations but requires large numbers of animals for studies to be powered to see differences between treatments. Rodents tend to not be very good models for carotenoid absorption and distribution since they do not absorb carotenoid intact unless they are fed at supra-physiological doses^22^. In this study, the dose of tomato given to the mice was chosen to be sufficient to produce plasma lycopene levels that are consistent with humans consuming a diet rich in lycopene containing foods. Studies in free-living humans have shown a range of lycopene in plasma, from 200–1000 nmol/L^23, 24^. Here, continued consumption of diets containing 10% by weight tomato powder, mice reached 250–500 nmol/L lycopene. This again provides additional evidence that carotenoid bioavailability and metabolism differs between mice and humans.

Despite three times higher concentrations of phytoene in the *tangerine* tomato diet, lycopene was present at 10 fold higher levels in skin, and 2–4 fold higher concentrations in plasma. This provides rationale to feeding *tangerine* tomatoes when increasing tissue lycopene is desirable, and contrary to others who have found that phytoene is more bioavailable than lycopene^25, 26^.

### Tumor incidence

**Figure 1:**
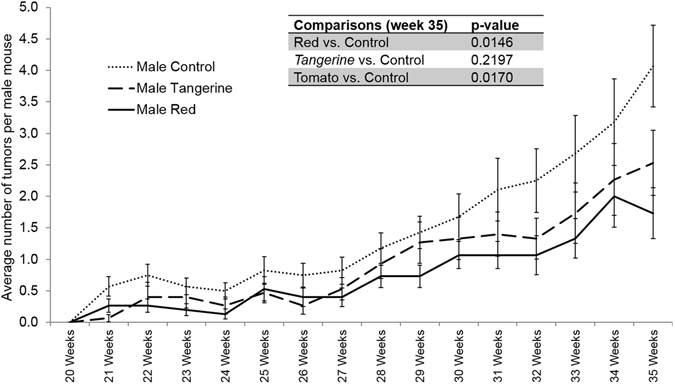
Tumor number progression in male mice fed control diets (dotted line) vs. tangerine tomato diets (dashed line) vs. red tomato diet (solid line). Significant differences exist at end-of-study between animals on red vs. control diets, and on tomato (both diets pooled) vs. control diets.

To our knowledge, this is the first study investigating the effects of tomato consumption on keratinocyte carcinomas *in vivo*. Tumor number from 20 to 35 weeks can be seen in (Fig. 1). Numbers below are means ± SEM. Male mice on control diets developed significantly more tumors (4.04 ± 0.65 tumors) than male mice on red tomato diets (1.73 ± 0.50 tumors, *P* = 0.015). Male mice on control diets developed significantly more tumors than mice on tomato-containing diets (i.e. both red and *tangerine* tomato fed mice combined, 2.03 ± 0.45 tumors, *P* = 0.017). These data provide convincing evidence that tomato consumption, in a model of disease prevention, can modulate risk for cutaneous squamous cell carcinomas. Male mice on control diets were not significantly different from male mice on *tangerine* diets (2.36 ± 0.50 tumors, *P* = 0.2197). There were no significant differences in tumor number for any of the female mice. Previous studies have demonstrated that after exposure to UVB, male mice develop tumors earlier, and these tumors are more numerous, larger and more aggressive than in female SKH-1 mice^27^. These changes may also be attributable to lower antioxidant levels, more cutaneous oxidative DNA damage and increases in GR-1^+^CD11b^+^ myeloid cells in males^27, 28^. We hypothesize that because the overall number of tumors were small, differences were not noted between diet groups in female mice, though this would require additional experiments to demonstrate. No significant differences of tumor grade were found among any of the treatment groups. Feeding 10% tomato containing diets has been shown to decrease tumor development at other sites, including prostate^29, 30^. Previous work has demonstrated that feeding a 10% *tangerine* tomato containing diet can decrease UV-induced production of cyclobutane pyrimidine dimers, myeloperoxidase activity and p53 positive epidermal cells in male SKH-1 mice, suggesting that tomatoes may act via reducing inflammation and subsequent DNA damage in the skin^14^. It has also recently been shown that administration of a lycopene rich tomato nutrient complex could be protective against UVA1 (longwave UV radiation) and associated alterations in gene expression^31^. Other compounds (including melatonin and vitamin D metabolites) have also been demonstrated to be protective against UVB light^32, 33^.

### Untargeted metabolomic analyses

Each carotenoid seems to have little relationship between concentration in the plasma and skin after linear regression (R^2^ < 0.1) suggesting, in this model, that skin carotenoid analysis would be necessary to understand concentrations in the skin itself. Additionally, when regressing tumor number with skin or plasma carotenoid concentrations, little relationship is apparent (i.e., mice with higher plasma or skin carotenoids do not seem to have fewer or smaller tumors). This lead us to conduct untargeted metabolomic analyses of murine skin, to better understand the global differences in small molecular weight metabolites present, without any preconceived bias.

Using untargeted metabolomics allows global profiling of differences between groups; in this case, the skin of animals fed control or tomato-containing diets. After removal of compounds present in processing blanks, full scan MS data collection on methanol extracts yielded over 8000 entities, with the stipulation that each entity must be present in every sample in at least one group. After conducting moderated t-tests (between animals on control vs. tomato diets) or ANOVA (comparing animals on control vs. *tangerine* tomato vs. red tomato) with Tukey’s post-hoc test and applying the Benjamini-Hochberg false discovery rate multiple testing correction, 17 entities achieved statistical significance. Of these 17 compounds, 10 can be definitively or tentatively identified as derived from tomato glycoalkaloids (Table 4).

**Table 4:** Metabolites in murine skin that differentiate animals on control vs. tomato diets linked to tomato glycoalkaloids. All compounds listed are absent in control animal skin and present in skin of animals on both *tangerine* and red tomato diets.

Retention time (min)	Tentative identification^a^	Monoisotopic mass	Observed mass [M + H]	ppm	Molecular formula
6.13	Dihydroxy-tomatidine isomer 1	447.3349	448.3420	1.48	C_27_H_45_NO_4_
6.37	Dihydroxy-tomatidine isomer 2	447.3349	448.3415	1.70	C_27_H_45_NO_4_
6.60	Dehydro-dihydroxytomatidine	445.3192	446.3249	3.82	C_27_H_43_NO_3_
6.97	Dihydroxy-tomatidine isomer 3	447.3349	448.3414	2.15	C_27_H_45_NO_4_
7.11	Dihydroxy-tomatidine isomer 4	447.3349	448.3418	1.70	C_27_H_45_NO_4_
7.13	Hydroxy-tomatidine isomer 1	431.3399	432.3463	1.96	C_27_H_45_NO_3_
7.22	Didehydro-hydroxytomatidine	427.3086	428.3152	2.57	C_27_H_41_NO_3_
7.43	Hydroxy-tomatidine isomer 2	431.3399	432.3468	3.12	C_27_H_45_NO_3_
7.72	Tomatidine^b^	415.3450	416.3517	2.24	C_27_H_45_NO_2_
7.94	Dehydrotomatidine (tomatidenol)	413.3294	414.3357	3.34	C_27_H_43_NO_2_

^a^Identities assigned via accurate mass, relative retention time and MS/MS fragmentation patterns. ^b^Tomatidine identity confirmed by authentic standard.

Tomatitidine, the aglycone of tomato glycoalkaloid α-tomatine, was confirmed to be present via authentic standard. Both tomatidine and α-tomatine have been shown to be anti-carcinogenic *in vitro* ^34, 35^ and have *in vivo* biological effects (including inhibiting skeletal muscle atrophy and weakness^36, 37^ and reducing plasma lipoprotein concentration^38^), lending plausibility to the idea that these compounds could be at least partially responsible for the decreases in tumor number observed here. Other identities can be tentatively assigned via accurate mass, relative retention time and MS/MS fragmentation patterns. This represents the first report of derivatives of tomato glycoalkaloids *in vivo*, as these compounds were previously thought to not be absorbed^39, 40^. The lack of absorption of tomato glycoalkaloids was the proposed reason for their lack of toxicity observed in populations consuming high glycoalkaloid tomatoes^41^.

## Conclusions

Overall, male mice that consumed tomato-containing diets developed fewer UVB-induced skin tumors compared to male mice that did not consume tomatoes. Highly sensitive HPLC-DAD-MS/MS methods were developed to identify and quantify carotenoids in both murine plasma and skin. SKH-1 male and female mice consuming a *tangerine* tomato powder containing diet accumulate increased levels of lycopene in their plasma and skin despite there being about three times less lycopene in the *tangerine* tomato diet as compared to the red tomato diet. This further confirms an increase in bioavailability of lycopene from *tangerine* tomatoes. Additionally, the concentrations of carotenoids in the skin of humans are similar to the concentrations determined in these tomato fed SKH-1 mice. Tomato alkaloids, including tomatidine, have also been documented as present in the skin of animals consuming tomato-containing diets suggesting they may be compounds responsible for the tumor number decrease noted in this study. These data suggest the need for further studies investigating the role that tomatoes and tomato phytochemicals play in the mediation of keratinocyte carcinomas.

## Methods

### Reagents

Optima grade water and methanol, and HPLC grade hexanes, ethanol, acetone, toluene, methyl *tert*-butyl ether (MTBE) and dichloromethane were purchased from Fisher Scientific (Pittsburgh, PA, USA). Ammonium acetate was from J.T. Baker (Phillipsburg, NJ, USA), butylated hydroxytoluene (BHT) and tomatidine hydrochloride were from Sigma-Aldrich (St. Louis, MO, USA). All-*trans*-lycopene was crystallized from tomato paste^42^ and phytoene, phytofluene, ζ-carotene, neurosporene and tetra-*cis*-lycopene were isolated from *tangerine* tomatoes using preparative HPLC and purity was tested, for use as external standards for quantitation^15^.

### Animal diets

Red and *tangerine* tomatoes (*Solanum lycopersicum*) were grown at the OSU North Central Agricultural Research Station in Fremont, OH. *Tangerine* (high in *cis*-lycopene) varieties FG04-167, FG04-169 and 7531, and red variety PS696, were harvested and delivered to The Ohio State University Food Industries Center, where the tomatoes were immediately diced and frozen, and stored at −40 °C. The tomatoes were then freeze dried in a Virtis Ultra 35LE lyophilizer (SP Industries/SP Scientific, Warminster, PA, USA). The dry tomato tissue was then pulverized in a vertical cutter mixer (Hobart, Troy, OH, USA) and stored in vacuum sealed plastic bags in the dark at −20 °C. These dry tomato powders were sent to Research Diets, Inc. (New Brunswick, NJ, USA) to be incorporated at a 10% (w/w) level into a modified AIN-93G base feed^14^ and pelleted. The control feed contained corn starch and dextrose in place of tomato powder to adjust to a matched macronutrient composition^29^. All feed was stored at −20 °C throughout the duration of the study, until it was weighed and distributed to the mice cages. The feed was replaced in the animal cages every 2–3 days to minimize carotenoid degradation.

### Animal study design

Four week-old male and female outbred SKH-1 hairless mice (Charles River Laboratories, Wilmington, MA, USA) were housed in a vivarium at The Ohio State University according to the requirements established by the American Association for Accreditation of Laboratory Animal Care. All the experimental treatment procedures were approved by The Ohio State University’s Institutional Animal Care and Use Committee (IACUC) before the initiation of any studies (IACUC Animal approval protocol #2010A00000083). Mice were fed control diets, a 10% *tangerine* tomato powder diet or a 10% red tomato powder diet.

**Figure 2:**
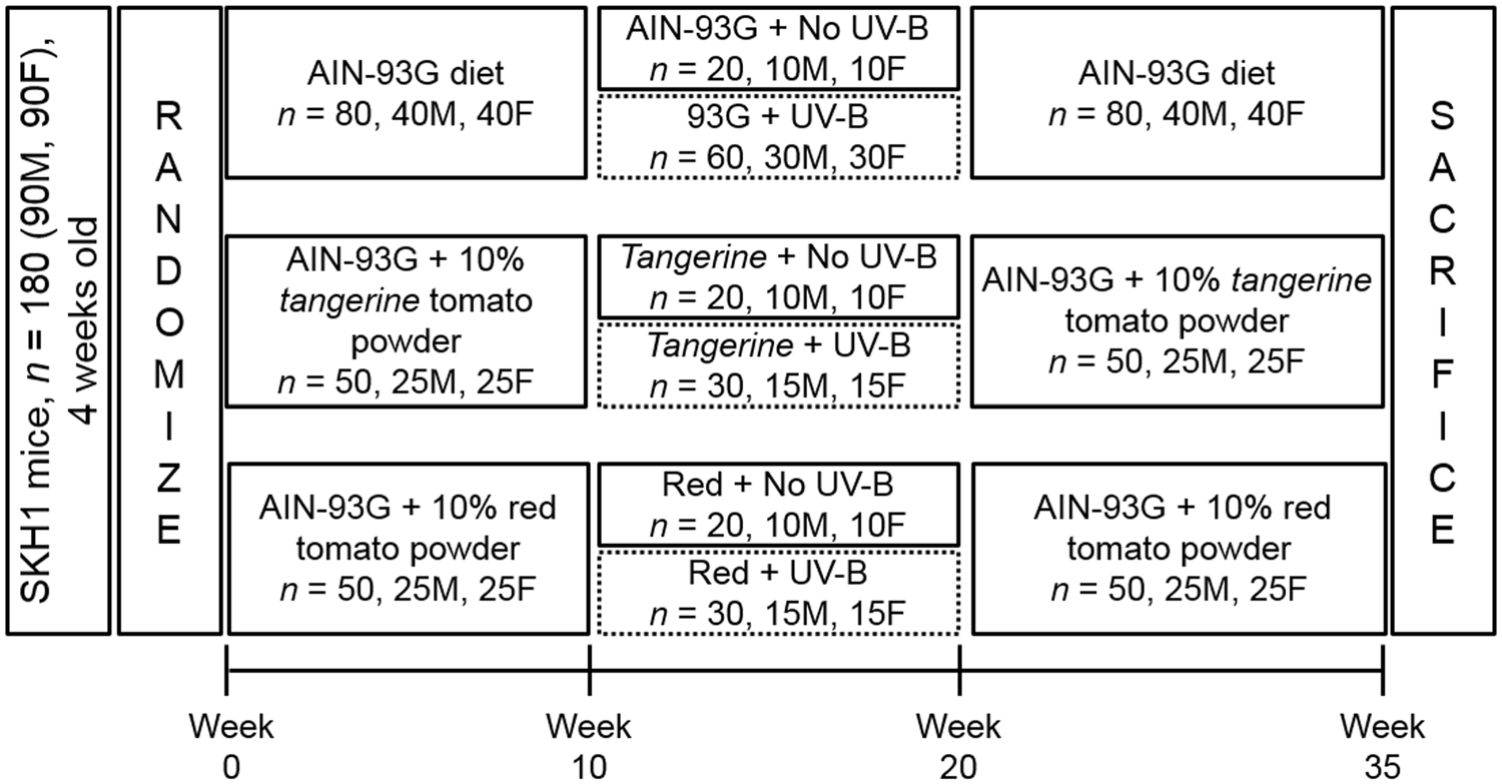
Simplified schematic of animal study design.

Mice began on their specified diets upon receipt and remained on their diets for the duration of the 35-week long study. At the beginning of week 11 through week 20, mice on their respective diets were dorsally exposed to 2240 J/m^2^ UVB, previously determined to be 1 minimal erythemal dose (M.E.D., the dose required to induce reddening of the skin 24 hours after exposure), three times weekly on non-consecutive days^43^ (Fig. 2). UVB dose was calculated using a UVX radiometer and UVB sensor (UVP, Upland, CA) and delivered using Philips TL 40 W/12 RS SLV UVB broadband bulbs emitting 310–320 nm UVB light (American Ultraviolet Company, Lebanon, IN). During weeks 21–35, mice continued on their respective diets, without any additional UV exposure. This treatment protocol induces papilloma growth beginning at 6–10 weeks in males and 10–12 weeks post-UVB exposure in females, and culminates in squamous cell carcinoma development at approximately 25 weeks. Tumor number and size were measured weekly starting at week 18. Age and sex matched control animals were fed each of the diets but not exposed to UVB radiation. Dorsal mouse skin and blood plasma were harvested and snap frozen in liquid nitrogen immediately after sacrifice for further analyses. All samples were stored at −80 °C until extraction.

### Measuring skin fold thickness

Skin fold thickness, as measured using digital calipers, was used to measure inflammatory response and edema 48 hours after the first UV-B exposure^44^. Forty-eight hours after the first UVB exposure has been found to be the time of peak inflammation.

### Monitoring tumor progression

Tumors were tracked by 2 dimensional measurement by the same individual using digital calipers once per week at the onset of visible tumors^44^. After sacrifice, tumors from each mouse receiving UVB treatment were harvested and underwent histological grading^27^. Hematoxylin and eosin stained tissue were graded, in a blinded manner, by a board-certified veterinary pathologist. Grades of hyperplasia, papilloma (grades 1–3), microinvasive squamous cell carcinoma (grades 1–3) or full invasive squamous cell carcinoma were assigned^45^. The histological features of each tumor have been described^45^, and representative macroscopic photographs published^46^.

### Extraction of carotenoids from murine skin

All extractions were performed in the dark, and samples were handled quickly to prevent degradation of carotenoids during extraction. Mouse skin was extracted using a method developed specifically for these samples^14^. Briefly, ~300 mg of frozen skin tissue was crushed using liquid nitrogen in a tissue pulverizer (The Cellcrusher, Cellcrusher, Cork, Ireland) to form a powder. The resulting powder was transferred in liquid nitrogen and weighed into glass centrifuge tubes. Water (1 mL) and ethanol with 0.1% butylated hydroxytoluene (1 mL) were added to the pulverized skin and the slurry was vortexed for 15 sec. Next, a 5:1 hexane:dichloromethane (DCM) solution (5 mL) as added, and samples were probe sonicated for 8 sec and then centrifuged for 5 min at 1000×*g*. The upper non-polar layer was removed, and the samples were re-extracted with 5 mL additional hexane/DCM solution. Extracts were then pooled and evaporated to dryness under nitrogen gas. Samples were stored at −80 °C until analysis via HPLC-DAD-MS/MS, within two days.

### Extraction of carotenoids from murine plasma

Plasma carotenoids were extracted modifying volumes for a small sample size from a previously described method^15^. Briefly, 200 μL of plasma was added to 200 μL of ethanol with 0.1% BHT. The sample was vortexed for 15 sec and 2 mL of 10:6:7:7 v/v/v/v hexane:ethanol:acetone:toluene (HEAT) was added, vortexed and centrifuged for 5 min at 1000× *g*. The top layer was then removed and extracted once more with HEAT and 0.5 mL saturated NaCl and the organic layers were pooled and evaporated to dryness under nitrogen gas. All extracts were stored at −80 °C prior HPLC-DAD-MS/MS analysis, within two days.

### High performance liquid chromatography tandem mass spectrometry (HPLC-DAD-MS/MS) of murine skin and plasma extracts for carotenoids

Extracts of skin tissue and blood plasma were separated using two separate reversed phase HPLC-DAD-MS/MS methods. For each, an Agilent 1260 HPLC (Santa Clara, CA) was interfaced with a QTRAP 5500 mass spectrometer (AB SCIEX, Carlsbad, CA) using an atmospheric pressure chemical ionization (APCI) operated in positive ion mode. Extracts of skin tissue were separated using a Sunfire™ 4.6 × 150 mm, 5 μm pore size, C18 column (Waters Corp, Milford, MA, USA) at 35 °C. Solvents A: 80:18:2 methanol:water:2% (w/v) aqueous ammonium acetate, and B: 78:20:2 MTBE:methanol:2% (w/v) aqueous ammonium acetate was used to separate the carotenoids of interest using the following linear gradient: initial conditions at 40% B to 63% B over 15 min, to 100% at 17 min and return to initial conditions for 3 min, for a total run time of 20 min. The flow rate was 1.3 mL/min and injection volume was 10 μL. Carotenoids were monitored as multiple reaction monitoring products of *m*/*z* as follows: phytoene 545 > 463, 421, 395, 327; phytofluene 534 > 461, 393, 325; ζ-carotene 541 > 457, 349, 271; neurosporene 539 > 457, 415, 389; lycopene 537 > 455, 269, as well as using DAD. Other MS parameters were the same as previously published^15^ with the exception of a declustering potential of 100 V. Dwell times were maximized to maintain 15 data points across each peak. Standard reference materials were created from *tangerine* and red tomato extracts to monitor and correct for MS response throughout sample analysis. Phytoene, phytofluene, ζ-carotene and tetra-*cis*-lycopene in plasma were quantified with photodiode array detection, and neurosporene, other-*cis*-lycopene and all-*trans*-lycopene were quantified using a sum of the MS/MS transitions. Extracts from murine plasma were analyzed using methods previously reported^15^.

### Targeted statistical data analysis

Statistical analysis was conducted within SAS® v. 9.3 (SAS Institute Inc., Cary, NC, USA) with α = 0.05 and statistical significance when *P* < 0.05. Animal weights and skinfold thickness were analyzed using Student’s t-test (between sexes and UV status) and ANOVAs with Tukey’s post hoc test (between diets). Univariate models were fit to understand the effects of UV status, sex and tomato type and their two-way interactions on each carotenoid concentration in both the plasma and the skin. Tumor number differences at the end-of-study were assessed using the Wilcoxon rank sum test due to non-normal distribution of data.

### Untargeted metabolomics sample preparation from mouse skin

Dorsal skins of male mice fed control, *tangerine* and red tomato diets, but not exposed to UV were randomly chosen and extracted using polar solvents for subsequent metabolomic analyses (n = 6 per group). Skin was cut into 2 × 2 mm squares and 100 mg was placed into a tissue homogenizing tube with ceramic beads designed to homogenize skin samples (CKMix, Precellys lysing kit, Bertin Technologies, France) with 1 mL cold methanol. The tubes were then inserted into a Precellys24 (Bertin Technologies) tissue homogenizer and extracted 3 times at 6,500 rpm, each for 20 sec with 10 min waiting time (on ice) between each extraction. The extraction tubes were centrifuged (2000× *g*) to pellet the ceramic beads and 200 µL aliquots were dried down under nitrogen. Quality control samples were also made pooling equivalent amounts of extracts from all animals. All extracts were stored at −80 °C prior to analysis, within 24 h.

### Metabolomics data acquisition via UHPLC-QTOF-MS

Extracts of murine skin were re-dissolved in 1:4 methanol:water, centrifuged at 21,130× *g* and the supernatant filtered through PTFE filters (0.2 μm pore size, 4 mm diameter, EMD Millipore, Darmstadt, Germany) before analysis. Samples were run on an Agilent 6550 Q-TOF-MS with an Agilent 1290 UHPLC (Agilent Technologies, Santa Clara, CA, USA) equipped with an Agilent ZORBAX Eclipse Plus C18 RRHD (2.1 × 150 mm, 1.8 μm) column at 50 °C. The samples were separated using a gradient of water (A) and acetonitrile (B), both with 0.1% formic acid added, at 0.4 mL/min. The gradient was as follows: 0% B, held isocratic for 2 min, linear gradient to 100% B over 10 min, held for 2 min, and reconditioned at 0% B for 2 min for a total run time of 16 min. Injection volumes were 2 μL. The TOF-MS with ion funnel was run with electrospray ionization in positive ion mode and collected data from 50–1700 *m*/*z*. The gas temperature was 150 °C, drying gas at 18 L/min, nebulizer at 30 psig, sheath gas temperature at 350 °C, and the sheath gas flow at 12 L/min. Additional MS/MS analysis was conducted in separate runs on the same instrument after statistical analysis was completed, to gather further structural information of compounds of interest.

### Metabolomics data processing and analysis

Peak finding and feature alignment of UHPLC-QTOF-MS data was conducted using Profinder B.07.00 (Agilent Technologies). Compounds present at 10-fold higher concentration in skin as compared to process blanks (solvent extracted in tissue homogenizer tubes without skin) were kept, and data was log2 normalized. Data visualization and statistical analysis was conducted within Mass Profiler Professional (Agilent Technologies) and MetaboAnalyst 3.0^47^.
